# Soil and crop data from a long-term organic fertilization trial in Sub-Sahelian market gardening

**DOI:** 10.1016/j.dib.2026.112456

**Published:** 2026-01-08

**Authors:** Marie-Liesse Vermeire, Pathé Basse, Samuel Legros, Falilou Diallo, Anne Desnues, Frédéric Feder

**Affiliations:** a CIRAD, UPR Recyclage et Risque, F 34398 Montpellier, France; b Recyclage et Risque, Univ Montpellier, CIRAD, F‑34398 Montpellier, France; c IESOL, IRD-ISRA, Bel-Air Centre, P.O. Box 1386, P.C 18524, Dakar, Senegal; d UCAD, FST, Department of Animal Biology, Dakar-Fann, 5005, Senegal; e IRD, UAR 191 IMAGO, Laboratoire des Moyens Analytiques de Dakar, Sénégal

**Keywords:** Sewage sludge, Poultry litter, digestate, SOERE PRO, Senegal

## Abstract

Recycling the growing stock of organic waste products (OWP) from cities, factories, and farms is a key challenge for sustainable agriculture. However, it must be done with awareness of performances but also potential long-term environmental and health risks. In this context, the SOERE PRO observatory was established ("Systèmes d'Observation et d'Expérimentation pour la Recherche en Environnement - Produits Résiduaires Organiques'', a label granted by the French National Research Alliance for the Environment (AllEnvi) to recognize high-quality research infrastructures, which translates to "Long-term Observation and Experimentation Systems for Environmental Research - Organic Waste Products''), including the trial in Sangalkam, in the Dakar region of Senegal, where these data are collected. Since 2016, four fertilizer types - one mineral (synthetic) and three organic - have been applied annually to three successive vegetable crops (tomato, lettuce, carrot). The dataset currently covers the period 2016 - 2025, with data collection ongoing and new data to be added in the future. Manual weeding and hoeing is carried out regularly for each crop, no pesticides are used for crop protection on the trial. A comprehensive, multi-variable dataset is consistently documented, including soil physico-chemical parameters measured annually at three depths, organic waste product characterization, crop yield and quality parameters, and detailed management activities, making it particularly suitable for process-based modelling and long-term impact assessment. The originality of this dataset lies in its long duration, the diversity of organic and mineral fertilization strategies, the inclusion of multiple vegetable crops per year, and its location under Sub-Sahelian conditions, a context for which long-term agronomic datasets remain scarce. All soil, OWP and vegetables samples are stored in a sample bank in Dakar, and available for additional analyses. The objective of this dataset is to provide long-term, integrated information on crop productivity, crop quality, and soil responses to repeated organic and mineral fertilization in a Sub-Sahelian market-gardening system. The dataset is publicly available through a Dataverse repository for free (re)use in meta-analyses, process-based modelling, and environmental studies, notably to improve understanding of nutrient cycling, contaminant dynamics, soil biodiversity, and long-term soil functioning in Sub-Sahelian agroecosystems, and to support sustainable land management and food security in Southern countries under future climate change.

## Graphical Abstract

**Figure:**
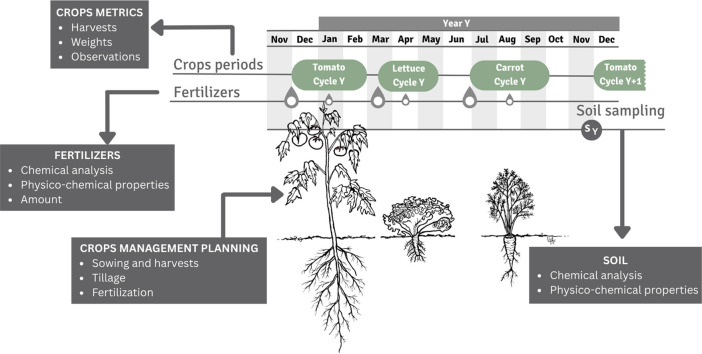

Specifications Table

**Table:** 

Subject	Earth & Environmental Sciences
Specific subject area	Long term field experiment testing the impact of the recycling of organic waste products in agriculture on soil and crops.
Type of data	Table (.xlsx format). Raw data.
Data collection	The data was collected on a long-term field trial. All management activities performed on the site, as well as applied doses of organic and synthetic fertilizers are recorded since 2016 (and still ongoing). Soil samples are collected routinely each year in November/December, at the end of the rotation, and OWP samples are collected before each crop. The samples are analyzed for physico-chemical parameters (AA3 Seal Analytical AutoAnalyzer, Thermo elemental analyzer, MP-AES Agilent microwave plasma atomic emission spectrometer). Several parameters are monitored routinely on the crops (one cycle of lettuce, carrot and tomato per year): crop yields, characteristics of the harvests, fresh and dry biomass measurement. Some additional measurements are also recorded for some years (pests and disease observations).
Data source location	• Institution: CIRAD • City/Town/Region: Sangalkam, Rufisque department, Dakar region • Country: Senegal • Latitude and longitude for collected samples/data: 14°47′34″, 17°13′30″
Data accessibility	Repository name: Dataverse Data identification number: 10.18167/DVN1/CHZ81W [1] Direct URL to data: https://doi.org/10.18167/DVN1/CHZ81W
Related research article	None.

## 1 Value of the Data

• This dataset provides one of the very few long-term, continuous time series available for intensive market-gardening systems under Sub-Sahelian conditions. Covering the period 2016 to 2025 (and still ongoing) with three crop cycles per year, it captures interannual variability that is rarely documented for vegetable crops in this region.
• The originality of the dataset lies in the combination of three vegetable crops (tomato, lettuce, carrot), four fertilization treatments (three organic waste products and one mineral control), two different doses, and repeated applications over multiple years, making it comparable to multiple long-term field trials conducted simultaneously within a single experimental system.
• The high temporal resolution of crop measurements, together with detailed records of management practices (organic inputs, mineral fertilization, tillage, and crop calendar) and soil physico-chemical properties measured annually at three depths, makes this dataset particularly suitable for the calibration and validation of soil–crop simulation models under semi-arid intensive horticultural conditions.
• The duration and completeness of the dataset allow part of the time series to be used for model calibration and another independent part for validation, which is rarely possible for tropical vegetable cropping systems amended with organic waste products.
• In addition, the dataset constitutes a well-documented historical reference for the physical soil, plant, and organic waste samples stored in the Sample Bank in Dakar, enabling future retrospective analyses (e.g. nutrient turnover, trace element accumulation) linked to clearly identified treatments and management histories.

## 2 Background

Recycling organic waste products (OWP) to soil has been practiced for millennia to maintain fertility. In the 20th century, it was often replaced by synthetic fertilizers (SF), which, while effective, contribute to greenhouse gas emissions, water eutrophication, and soil degradation [[1], [2], [3], [4], [5]]. Sustainable agriculture now encourages a return to organic fertilization, which improves soil biological functions, increases organic carbon, and enhances physical fertility [[6], [7], [8], [9], [10], [11]]. However, challenges remain. OWP nutrient composition (N, P, K) varies and often mismatches crop needs, making them less practical than SF with known compositions. They can also induce nitrogen leaching or volatilization and introduce contaminants (pathogen agents, organic or mineral) that may accumulate in soils and transfer to crops or animals [[12], [13], [14], [15]]. Social and logistical barriers might further limit adoption. Evaluating long-term OWP impacts is essential to confirm equivalent yields than SF, improved soil fertility, and acceptable environmental and sanitary risks. The SOERE PRO Senegal trial, launched in 2016 within the SOERE PRO observatory (INRAE, ALLENVI), addresses these questions [16]. It studies the agronomic effects and risks of repeated OWP applications, aiming to guide their sustainable use in agriculture.

## 3 Data Description

This article describes the dataset linked to the SOERE PRO Senegal field trial. It includes all management activities, as well as soil, OWP and plant analysis and observations since 2016. The data were collected from February 2016 and are updated regularly (the trial is still currently running). The SOERE PRO Senegal dataset is in English, in the Microsoft Excel format (version 16.97.2), and is spread over six separate files:

• **1_DATA_SOIL_SOERE-PRO_SENEGAL.xlsx**: containing the results of the soil physico-chemical analysis.
• **2_DATA_FERTILIZATION_SOERE-PRO_SENEGAL.xlsx:** containing the results of the OWP analysis, and the exact applied doses of raw matter (OWP and SF).
• **3_DATA_MANAGEMENT_SOERE-PRO_SENEGAL.xlsx**: containing all management activities (dates of shallow tillage, fertilization, sowing, transplanting, harvest, cycle length) at each field plot.
• **4_DATA_CROP_CARROT_SOERE-PRO_SENEGAL.xlsx**: presenting the harvest and crop observations data for the carrot crop.
• **5_DATA_CROP_LETTUCE_SOERE-PRO_SENEGAL.xlsx**: presenting the harvest and crop observations data for the lettuce crop.
• **6_DATA_CROP_TOMATO_SOERE-PRO_SENEGAL.xlsx**: presenting the harvest and crop observations data for the tomato crop.

Every file is self-explanatory and contains detailed information in the README sheet, including variable names, units and a description of the method used (including laboratory protocols). The structure of the dataset (file names, sheet names per file, and column names per sheet) are presented in Table 1.

**Table 1:** Content of the dataset: the name of each file, the sheets per file and name of columns in each sheet.

File name	Sheet name	Column names per sheet
1_DATA_SOIL_SOERE-PRO_SENEGAL	Readme	Column names; Description; Values/unit
	Data_SOIL	**Year, Cycle, Treatment, Plot**, Depth_soil, Date_sampling, Ref_SN, Clay, Fine_silt, Coarse_silt, Fine_sand, Coarse_sand, pH_H2O, pH_KCl, N_NO3, N_NH4, N_tot, C_tot, C_org, P_tot, P_avail, Ca_exch, Mg_exch, Na_exch, K_exch, CEC
2_DATA_ FERTILIZATION _SOERE-PRO_SENEGAL	Readme	Column names; Description; Values/unit
	Data_CHEM_PROPERTIES	**Year, Cycle, Fertilizer_type, Crop**, Date_sampling, Ref_SN, DM, pH_H2O, N_Kjeld, N_tot, C_tot, P_tot, K_tot, Ca_tot, Mg_tot, Na_tot
	Data_AMOUNT	**Year, Cycle, Crop, Fertilizer_type, Treatment**, Amount_applied_OWP, Amount_applied_chem_10_10_20, Amount_applied_chem_urea, Amount_applied_chem_K2SO4
3_DATA_MANAGEMENT_ SOERE-PRO_SENEGAL	Readme	Column names; Description; Values/unit
	Data_MANAGEMENT	**Year, Cycle, Crop**, Date_sowing, Date_transplanting_or_thinning, OWP_application_date, SF_application_date, Date_chemical_complementation, Harvest_date, Cycle_duration, Tillage_start_date, Tillage_end_date
4_DATA_CROP_CARROT_SOERE-PRO_SENEGAL	Readme	Column names; Description; Values/unit
	Data_CARROT_harvest	**Year, Cycle, Treatment, Plot, Cropping**_**bed**, Tot_nb_carrots, Tot_weight_carrots, Nb_diam_sup_2cm, Weight_diam_sup_2cm, Nb_diam_inf_2cm, Weight_diam_inf_2cm, Nb_deformed, Weight_deformed, Nb_damaged, Weight_damaged, Total_weight_leaves
	Data_CARROT_dry_weight	**Year, Cycle, Treatment, Plot**, Nb_carrots_in_sample, Carrot_root_tot_sample_fresh_weight, Carrot_root_tot_sample_dry_weight, Perc_hum_carrot_root, Mean_dry_weight_per_carrot_root
	Data_CARROT_observations	**Year, Cycle, Treatment, Plot**, Sample_nb, Length_carrot, Diameter_carrot, Height_leaves, Powdery_mildew_leaves, Alternaria_disease_leaves, Nematodes_roots, Cuscuta_leaves
5_DATA_CROP_LETTUCE_ SOERE-PRO_SENEGAL	Readme	Column names; Description; Values/unit
	Data_LETTUCE_harvest	**Year, Cycle, Treatment, Plot, Cropping_bed**, Tot_nb_lettuce, Tot_weight_lettuce, Nb_headed, Weight_headed, Nb_non_headed, Weight_non_headed
	Data_LETTUCE_dry_weight	**Year, Cycle, Treatment, Plot**, Nb_lettuces_in_sample, Lettuce_leaves_tot_sample_fresh_weight, Lettuce_leaves_tot_sample_dry_weight, Perc_hum_lettuce_leaves, Mean_dry_weight_per_lettuce_leaves
	Data_LETTUCE_observations	**Year, Cycle, Treatment, Plot**, Sample_nb, Lettuce_diam_H-2week, Lettuce_diam_H-1week, Lettuce_diam_H, Leaf_miner, Brown_spots_leaves, Nematodes_roots, Root_necrosis
6_DATA_CROP_TOMATO_ SOERE-PRO_SENEGAL	Readme	Column names; Description; Values/unit
	Data_TOMATO_harvest	**Year, Cycle, Treatment, Plot**, Harvest_nb, Date_harvest, Nb_plants, Cat1_tot_weight, Cat1_tot_nb, Cat1_inf47mm_weight, Cat1_inf47mm_nb, Cat1_47_57mm_weight, Cat1_47_57mm_nb, Cat1_57_67mm_weight, Cat1_57_67mm_nb, Cat1_67_82mm_weight, Cat1_67_82mm_nb, Cat1_82_102mm_weight, Cat1_82_102mm_nb, Firmness, Cat2_weight, Cat2_nb, Cat3_weight, Cat3_nb, Cat4_weight, Cat4_nb, Cat5_weight, Cat5_nb
	Data_TOMATO_dry_weight	**Year, Cycle, Treatment, Plot**, Harvest_nb, Nb_tomatoes_in_sample, Tomato_fruit_tot_sample_fresh_weight, Tomato_fruit_tot_sample_dry_weight, Perc_hum_tomato_fruit, Mean_dry_weight_per_tomato_fruit
	Data_TOMATO_observations	**Year, Cycle, Treatment, Plot**, Plant_nb, Observation_date, TYLCV, Bacterial_wilt Alternaria_disease, Bacterial_leaf_spot, Helicoverpa_armigera, Tuta_absoluta, Aleyrodoidea, Mites, Mealybug, Nesidiocoris_tenuis, Liriomyza_sp, Aphids, Grasshopper, Thrips_sp, Geometridae_caterpillars, Spodoptera_sp

The dataset was deposited in the Cirad Dataverse platform on 06/06/2025, under the Creative Commons (CC BY-NC-SA 4.0) open license, under the name “A dataset of soil properties and crop observations from a long-term organic fertilization trial in Sub-Sahelian market gardening”, by Vermeire, Marie-Liesse; Pathe, Basse; Legros, Samuel; Falilou, Diallo; Desnues, Anne; Feder, Frédéric, with following DOI: https://doi.org/10.18167/DVN1/CHZ81W. The Cirad Dataverse is a trusted research data repository for datasets produced or co-produced by Cirad scientists and their partners, particularly in projects involving joint research units or collaborators from the Global South. It supports data sharing, publication, and long-term preservation in all file formats (preferably open/standard), with limits of 4 GB per file and 50 GB per dataset. Built on the open-source Dataverse software developed by Harvard’s IQSS, the platform is recognized by most publishers and is organized into collections managed by Cirad’s research units.

## 4 Experimental Design, Materials and Methods

### 4.1 Site location: main vegetable-growing region of Senegal

**Fig. 1:**
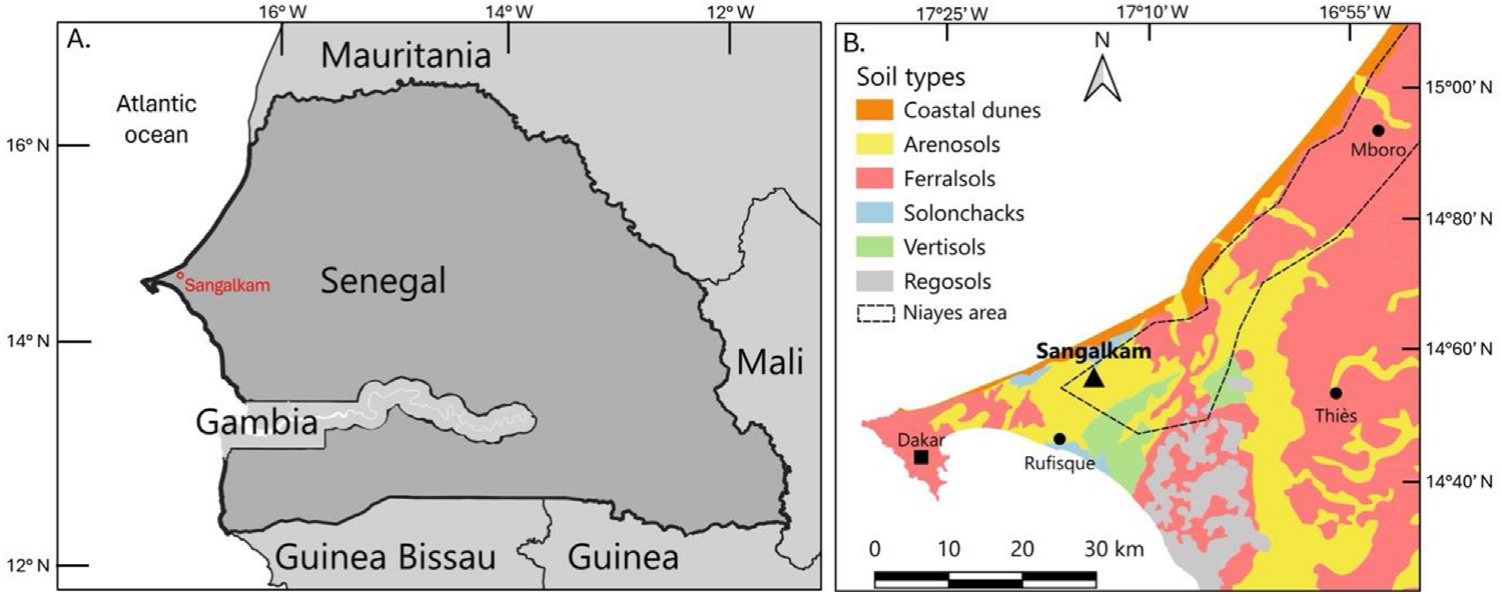
SOERE PRO Senegal field trial location and context. **A.** Sangalkam location within Senegal; **B.** Sangalkam area soil types (Source: [20]).

The long-term field experiment was initiated in 2016 at the Sangalkam experimental station of the Senegalese Institute of Agricultural Research (ISRA), located in the Rufisque department within the Dakar region of Senegal (14°47′34″ N, 17°13′30″ W; approximately 10 m above sea level). The station lies at the southern margin of the Niayes zone, which represents the main area for vegetable production in the country (Fig. 1).

The Niayes region extends over approximately 180 km along the Atlantic coast and varies in width from 5 to 30 km. It is characterized by a landscape of sandy dune systems interspersed with interdunal depressions, where shallow groundwater frequently reaches the surface and may cause seasonal flooding. The climate is classified as Sahelo-Sudanian, with a strong maritime influence driven by trade winds originating from the Azores High. Mean air temperatures range from 17 to 25 °C during the cooler season (December to April) and from 27 to 30 °C during the warmer months (May to November). Precipitation is concentrated in a short rainy season from July to October, with mean annual rainfall ranging between 300 and 600 mm [17].

The soil at the Sangalkam site is classified as an Arenosol [18,19] composed of Quaternary dune sands, representative of soils commonly found throughout the Niayes area. It is slightly acidic and characterized by low contents of organic matter, nitrogen, and phosphorus. Prior to the establishment of the experiment, the plot had been cultivated with cassava for two consecutive years. Following cassava harvest in September 2015, the soil was ploughed to a depth of 20 cm. At the onset of the experiment in February 2016, soil bulk density was 1.55 kg dm⁻³ in the 0 to 20 cm layer and 1.60 kg dm⁻³ in the 20 to 40 cm layer.

### 4.2 Experimental design and fertilization treatments

**Fig. 2:**
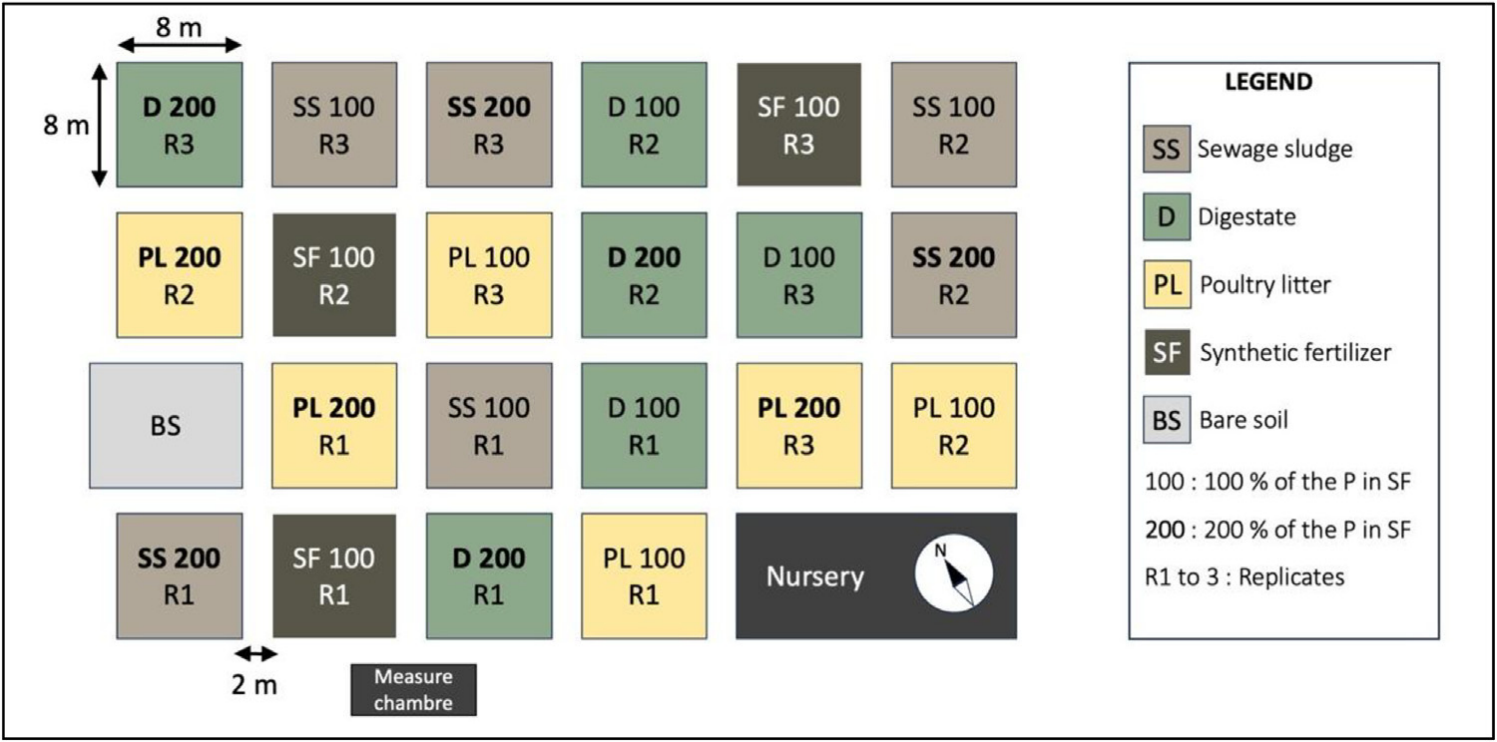
Spatial scheme of the experimental design of the SOERE PRO Senegal long-term field trial.

**Fig. 3:**
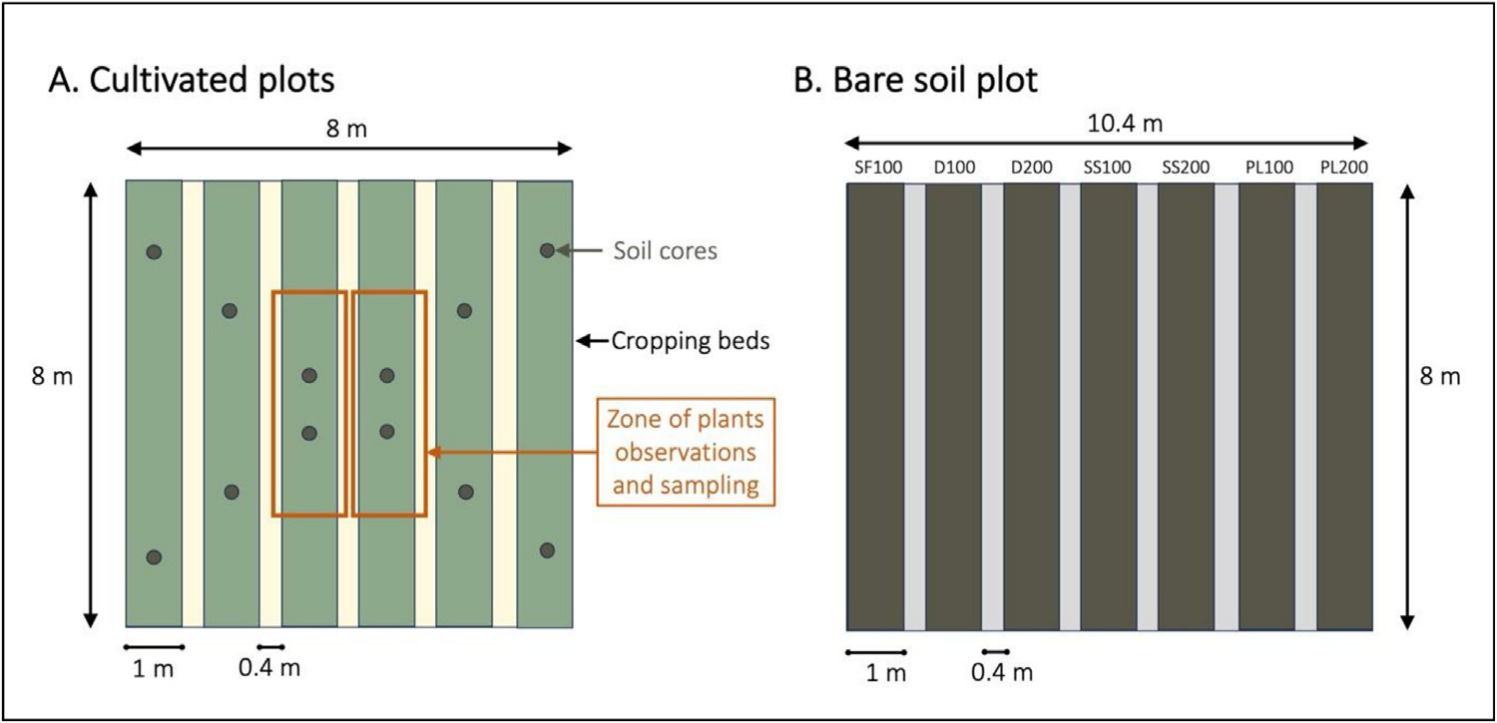
**A**. Organization of cropping beds within cultivated plots, and location of the soil sampling and of the zones for plant observations and sampling. **B.** Organization of the Bare Soil plot (BS), where no plant grew since 2016, and where the seven treatments are applied at the same time than on the cultivated plots.

The experimental layout comprises 22 square plots, each covering an area of 64 m² and separated from adjacent plots by 2 m (Fig. 2). Within each plot, six cultivation beds are established to reflect local market-gardening practices. Each bed measures 1 m in width and 8 m in length, corresponding to a surface area of 8 m², and beds are spaced 0.4 m apart (Fig. 3).

A fixed annual crop rotation is implemented, consisting of tomato, lettuce, and carrot grown sequentially each year. This rotation enables the assessment of fertilization effects on crops harvested for fruits, leaves, and roots, respectively. The experiment follows a completely randomized design with seven fertilization treatments; each applied to three replicate plots:

• **SF 100**: Synthetic fertilizer based on NPK (N-P_2_O_5_-K_2_O, 10–10–20), urea (CH_4_N_2_O, 46–0–0) and potassium sulphate (K_2_SO_4_, 0–0–51).
• **SS 100**: Dried sewage sludge at 100 % of the P needs.
• **SS 200**: Double the SS 100 dose.
• **PL 100**: Poultry litter at 100 % of the P needs.
• **PL 200**: Double the PL 100 dose.
• **D 100**: Anaerobic digestate from cow manure at 100 % of the P needs.
• **D 200**: Double the D 100 dose.

In addition to the cropped plots, a bare-soil control is included to evaluate treatment effects in the absence of vegetation. This bare-soil unit covers a total surface area of 83.2 m² and contains seven beds of 8 m² each. All seven fertilization treatments are applied simultaneously on this plot, with one treatment assigned to each individual bed (Fig. 3).

The fertilization is designed to correspond to the doses recommended by technical institutes in Senegal for each crop and yield that can be targeted in the zone (Table 2). The SF is applied in two stages to minimise nitrogen losses through volatilization. All of the crop P requirements are applied (NPK form) just after the lettuce or tomato plants are transplanted and the carrots are thinned. A supplement of N and K (N with urea - 46 % of N; and K with potassium sulphate −42 % of K) is applied in the middle of the cycle of the corresponding crop.

**Table 2:** Crop needs in N, P and K, and targeted yield.

Crop needs	N	P	K	Targeted Yield
	Kg.ha^-1^			T.ha^-1^
**Tomato**	120	39	175	50
**Lettuce**	120	28	200	50
**Carrot**	110	35	225	45

The OWP fertilization is calculated based on the N, P and K concentrations measured in the local poultry litter, digestate and sewage sludge (please refer to the section “OWP sampling and analysis”). These concentrations are converted into SF-equivalent, using the substitution rates (SR) presented in Table 3. The dose of raw OWP applied per plot is calculated to provide the same amount of SF-equivalent P than in the SF treatment (Eq. (1)). The organic treatments are applied three to five days before the lettuce and tomato plantlets are transplanted or the carrot seeds are sown. The treatments are applied to the soil surface before each crop and never buried. The gap between the SF-equivalent in N and K provided by OWP and the crop needs in N and K is filled by a supplement of N with urea and K with potassium sulphate, applied in the middle of the crop cycle. All crop residues as well as plant material from weeds are exported from the plots, making the OWP the only organic material applied on the plots. Note that the digestate treatment could not be used on the lettuce and carrot crops in the first campaign due to non-availability. In addition, the digestate showed variable properties and was often too liquid to provide nutrients in the desired quantities.

(1) $$\text{OWP}\;\text{quantity}\;\text{to}\;\text{satisfy}\;P\;\text{needs}\;\left(\frac{t}{\text{ha}}\right)=\;\frac{\text{crop}\;P\;\text{needs}\;\left(\frac{\text{kg}}{\text{ha}}\right)}{\text{OWP}\;P\;\text{content}\;\left(\frac{\text{kg}}{t}\right)\times \;\text{SR}-P}$$

**Table 3:** Substitution rate (SR) of nitrogen, phosphorus, potassium from synthetic fertilizers by organic waste products (OWP).

OWP	SR-N	SR-P	SR-K
Poultry litter	0.6	0.65	1
Sewage sludge	0.45	0.6	1
Digestate	0.7	1	1

### 4.3 Management

**Fig. 4:**
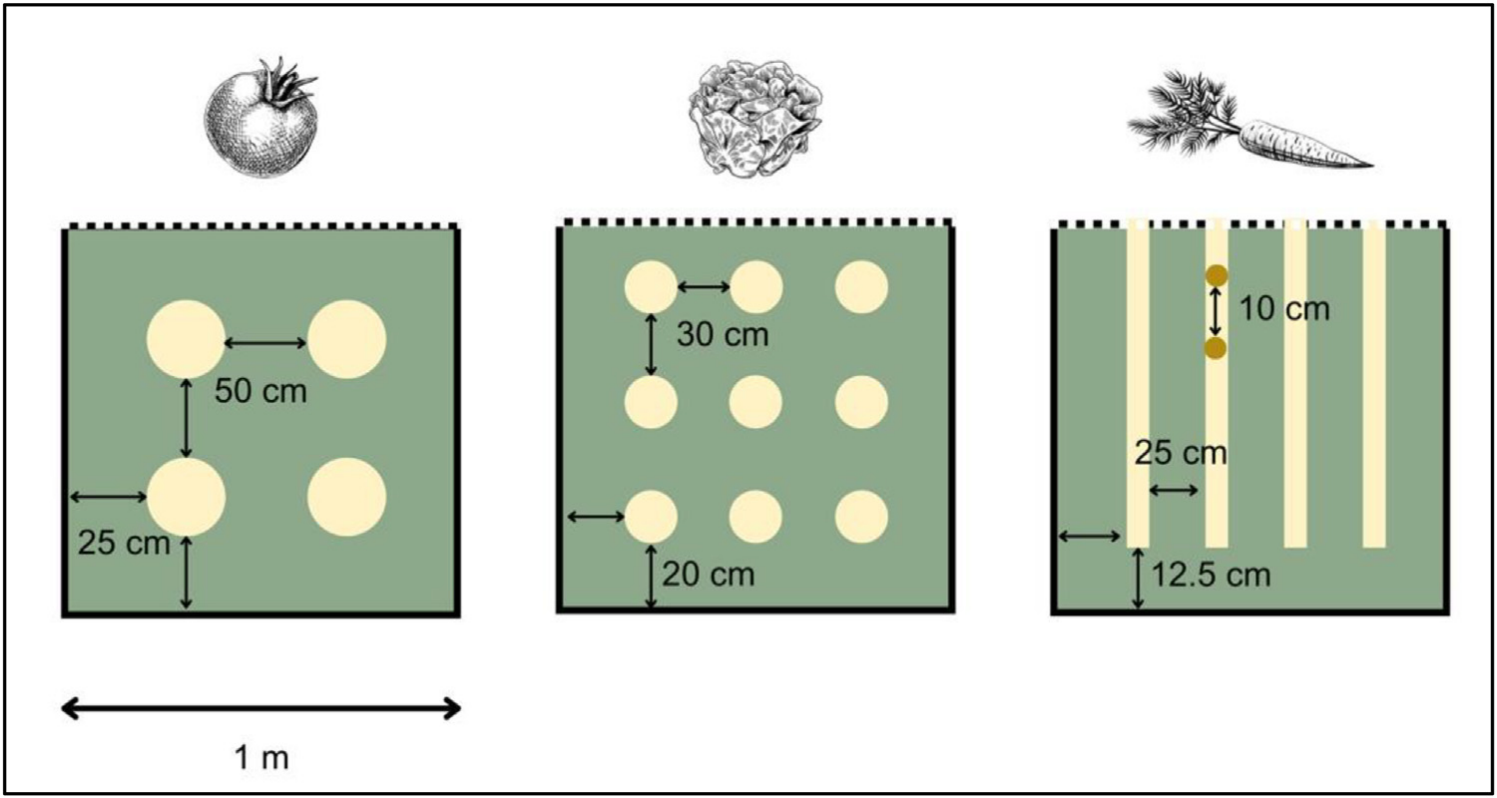
Row and inter-row spacing for the three crops cultivated per year (tomato, lettuce, carrot) in the cropping beds (6 cropping beds per plot).

Before every crop, a shallow manual tillage and levelling of the plots is performed. The lettuce and tomato plants are sown in a nursery before being transplanted to the experimental plots (25 days after sowing). After transplanting, regular observations are carried out during the first ten days to replace any dead plants. The spacing between lettuce plants is 30 cm, arranged in three rows parallel to the length of the beds (Fig. 4). The spacing of tomato plants is 50 cm, arranged in two rows parallel to the length of the beds (Fig. 4). Tomato plants are staked using wooden stakes approximately one meter tall, about 20 - 25 days after transplanting. The sowing of carrots is done directly within the plots (approximately 5 g of seeds per bed). Furrows spaced by 25 cm are opened in each bed using a row marker, resulting in four furrows parallel to the length of the beds (Fig. 4). One month after carrot seeding, thinning is performed to adjust spacing between carrot plants to 5–10 cm. Irrigation is carried out daily, at a rate of approximately 45 - 55 l per bed (8 m^2^) for all of the crops. Manual weeding and hoeing (to remove weeds) is carried out regularly for each crop, no pesticides are used for crop protection on the trial.

### 4.4 Soil sampling and analysis

Soil samples are collected routinely each year in Novembre or Decembre, at the end of the rotation. In each plot, composite samples composed of 12 soil samples (Fig. 3) are collected using an auger (diameter 5 cm) at three depth levels: 0–10, 10–20 and 20–40 cm depth. The samples are mixed and homogenized by passing them through a 2 mm sieve, and then air-dried, for analyses and storage. An additional soil sampling was performed in February 2016, to characterize the initial properties of the soil, with three composite samples per plot: 0–20, 20–40, and 40–60 cm. The physicochemical parameters are measured using the methods recommended by AFNOR (French Standardization Association). All analyses are carried out at the IMAGO service unit of IRD in Dakar. The particle size determination (five soil fractions: clay, fine silt, coarse silt, fine sand, coarse sand) is obtained by sampling with a Robinson pipette using Stockes' law for the clay and silt fractions, and by sieving for fine and coarse sand fractions. Soil pH is measured in a 1/2.5 vol ([soil/distilled water] for pH H_2_O and [soil/1 N KCl solution] for pH KCl) mixture measured by glass electrode. Mineral forms (available) of nitrogen are extracted with a 1 M KCl solution and assayed by colourimetry using a AA3 Seal analytical auto analyzer (Griess and Nessler methods). Total nitrogen determination is based on combustion of the sample under oxygen at 900 °C, with quantitative conversion of the released nitrogen compounds to N₂ via oxidation and reduction tubes, using a Thermo elemental analyzer (Dumas method). Total carbon determination is based on combustion of the sample under oxygen at 900 °C, with quantitative conversion of the released carbon compounds to CO₂ via oxidation and reduction tubes, using a Thermo elemental analyzer (Dumas method). For organic carbon determination, soil is oxidized at 135 °C with a mixture of concentrated sulfuric acid and 3 % potassium dichromate. The Cr^3+^ ions formed, proportional to the quantity of oxidized carbon, are measured by colorimetry using an AA3 AutoAnalyzer. Total phosphorus is determined after a hot digestion at 110 °C with aqua regia for five hours in mineralizer by colorimetry using an AA3 Seal Analytical AutoAnalyzer (Murphy and Riley method). Plant-available phosphorus is extracted with a solution of sodium bicarbonate 0.5 M. The extract is analyzed by an AA3 Seal Analytical AutoAnalyzer (Olsen method). Exchangeable calcium, magnesium, sodium and potassium are extracted with ammonium acetate 1 M at pH 7 and measured using a MP-AES Agilent (microwave plasma atomic emission spectrometer). Cation exchange capacity is determined by colorimetric analysis (AA3 Seal Analytical AutoAnalyzer) of ammonium fixed on exchange sites during the extraction of exchangeable cations, then displaced by a potassium nitrate solution 1 M.

### 4.5 OWP origin, sampling and analysis

**Fig. 5:**
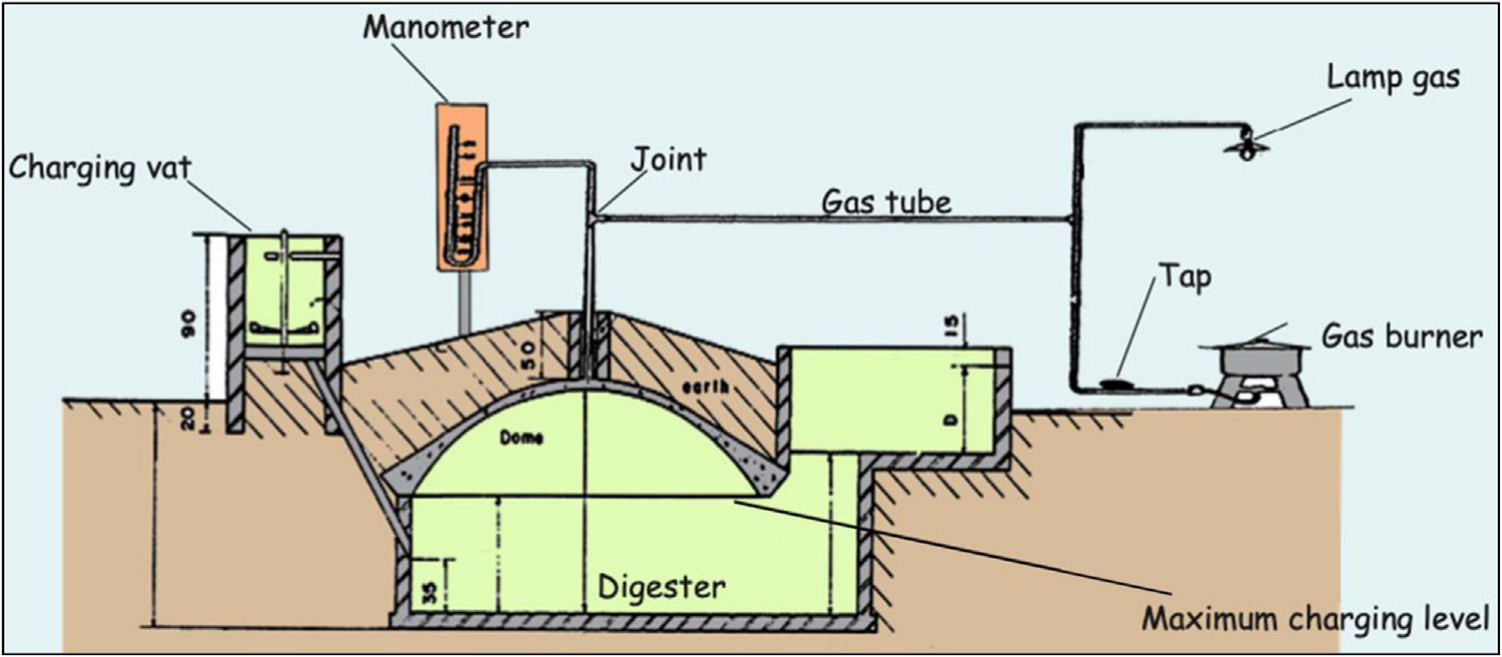
Design of the biodigester installed at the Station of Sangalkam (developed by the national biogas program of Senegal) (© Joseph Akigo).

Sewage sludge from 2016 to 2022 comes from the Cambérène treatment plant, located in the Pikine department (24 km from Sangalkam), then after 2022 from the Pikine treatment plant. At these facilities, sludge is first pumped from the bottom of the primary settling tank into the primary digester, where it is digested. Through overflow from the primary digester, the sludge flows into a secondary digester where it is further stabilized before being transferred to drying beds. Once dried, the sludge forms large, hard clumps that are used on the trial after light crushing. Poultry litter comes from farms located in the commune. These farms typically use peanut shells as bedding material for laying hens. At the end of the rearing period, the mixture of bedding and hen manure is packed into sacks by farmers for sale. This PL has a fine texture and is ready for use. The digestate derives from the transformation of cow dung (collected from a farm in the village of Noflaye, located 1 km from Sangalkam) in the biodigester installed at the Sangalkam experimental station (Fig. 5). The biodigester is equipped with an inlet tank for cow dung and an outlet chamber where excess production is discharged and the digestate is collected. Forty days after loading, the digestate is available for crop fertilization. The digestate is stored in 120-liter barrels left open near the plots.

Before each crop, OWP samples are collected. Three aliquots of the raw matter are prepared: 1) one aliquot is destined to the laboratory for analysis; 2) a second is used to measure the dry matter content, by drying in an oven at 65 °C until a constant weight is achieved; 3) a third is stored in the Sample Bank (samples of sewage sludge and poultry litter are stored at room temperature, digestate samples are stored in the freezer at −20 °C). All analyses are carried out at the IMAGO service unit of IRD in Dakar. The percentage of dry matter is calculated using the ratio between dry weight and fresh weight, after drying to a constant weight at 65 °C is achieved. The pH of OWP is measured in a 1/2.5 vol [OWP/distilled water] mixture by glass electrode. The Kjeldal Nitrogen is determined by mineralization by digestion in concentrated sulfuric acid and salicylic acid, with a selenium-based catalyst, followed by the measurement of ammonium ions by colorimetry using an AA3 Seal Analytical AutoAnalyzer (Berthelot reaction). Total nitrogen determination is based on combustion of the sample under oxygen at 900 °C, with quantitative conversion of the released nitrogen compounds to N₂ via oxidation and reduction tubes, using a Thermo elemental analyzer (Dumas method). Total carbon determination is based on combustion of the sample under oxygen at 900 °C, with quantitative conversion of the released carbon compounds to CO₂ via oxidation and reduction tubes, using a Thermo elemental analyzer (Dumas method). Total phosphorus is determined after a hot digestion at 100 °C, with nitric acid 2 % for three hours in mineralizer, by colorimetry using an AA3 Seal Analytical AutoAnalyzer (Murphy and Riley method). Total Potassium, Calcium, Magnesium, and sodium content is determined after a hot digestion at 100 °C, with nitric acid 2 % for three hours in a mineralizer, using a MP-AES Agilent (microwave plasma atomic emission spectrometer).

### 4.6 Crops observations and yield

Several parameters are regularly monitored for lettuce and tomato during the crop cycles on pre-identified plants. Plant identification is carried out using wooden stakes fifteen days after transplanting. Monitored plants are selected from beds no. 3 and 4, leaving a 2-meter margin on each end of the beds to minimize edge effects (Fig. 3). At harvest (performed manually), crop yields are measured per cropping bed on the whole plot (6 beds × 8 m^2^). Weighing is performed immediately after harvest using a precision electronic scale. Characterization of the vegetables are also carried out at harvest for all crops. The observations and yield measurements are performed as follow, per crop:

#### 4.6.1 Lettuce

• **Diameter:** Lettuce diameter is determined by measuring the distance between the tips of the outermost leaves using a graduated ruler. The largest measurement is considered the plant’s diameter. These observations are made two and one weeks before harvest and at harvest, on the six pre-identified plants (the same that will be used for sampling and analysis).
• **Diseases and Pest Damage:** at harvest, diseases and pest damage are assessed (leaf miner, presence of brown spots on leaves, nematodes in roots, root necrosis).
• **Yield:** The lettuces are manually uprooted and shaken to remove as much soil as possible from the roots. Leaves and roots are separated at the collar before weighing the leaves. Lettuces are divided into two categories: **category 1** - headed and **category 2** - non-headed. Lettuces in each category are counted and weighed per bed, to determine the percentage and biomass per category. The lettuce yields can be calculated based on the total biomass of the leaves, combining all lettuce categories, the real number of plants per plot, and corrected by the theoretical planting density - 9.75 plants per m^2^ (78 lettuces per plot / 8 m^2^ per plot) - according to the Eq. (2). This yield calculation, based on average lettuce weight, can be used to reduce the bias of certain factors that can influence yields.
  (2) $$\text{Yield}\;\left(\frac{\text{kg}}{m^{2}}\right)=\;\frac{\begin{array}{c} \;\\ Biomass\;per\;plot\;\left(Kg\;fresh\;weight\right) \end{array}}{\text{Number}\;\text{of}\;\text{plants}\;\text{per}\;\text{plot}}\;\times \;\text{Theoretical}\;\text{planting}\;\text{density}$$

#### 4.6.2 Carrot

**Fig. 6:**
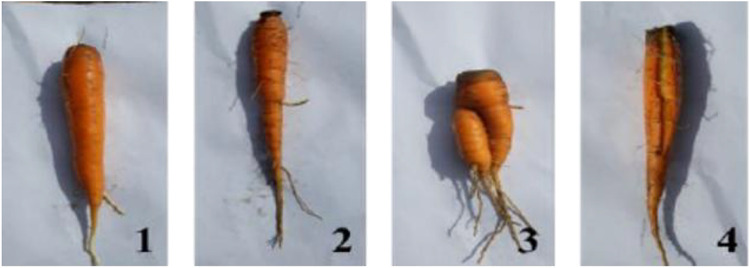
Pictures of the carrot categories.

• **Length and diameter of roots, height of leaves:** At the harvest, eight carrots are identified (four per central bed), to serve for observations and analysis. Carrot leaves and roots are separated at the collar. The length (up to pencil-size tip) is measured every year using a ruler and a calliper, collar diameter of roots and height of leaves for some years.
• **Diseases and Pest Damage:** at harvest, diseases and pest damage are assessed (powdery mildew on leaves, Alternaria disease on leaves, nematodes in roots, cuscuta on leaves).
• **Yield**: Carrot roots are manually uprooted and shaken to remove as much soil as possible from the roots. Carrot roots are divided into four categories based on their morphology (Fig. 6): **Category 1**: normal shape and a diameter greater than 2 cm; **Category 2**: normal shape and a diameter inferior than 2 cm; **Category 3:** deformed, twisted, or forked roots; **Category 4:** split, cracked, or damaged. The carrots in each category are counted and weighed per bed, to determine the percentage and biomass per category. The carrot yields can be calculated based on the total biomass of the roots, combining all carrot categories except for the rotten ones, the real number of plants per plot, and corrected by the theoretical planting density - 40 plants per m^2^ (320 carrots per plot / 8 m^2^ per plot) - and according to the Eq. (2). This yield calculation, based on average carrot weights, can be used to reduce the bias of certain factors that can influence yields, for example seed density per row or furrow depth.

#### 4.6.3 Tomato

• **Tomato Firmness:** Three tomatoes are selected per plot at each of the five harvests, and three measurements taken per tomato using a durometer. The selection of tomato samples is performed with a calibre-meter to select fruits of the same calibre and with a red/orange colour.
• **Diseases and Pest Damage:** Throughout the crop cycle and up to harvest, diseases and pest damage are assessed on specific dates, on the leaves and fruits of the eight pre-identified plants: 1) presence/absence of diseases: tomato yellow leaf curl virus (TYLCV), bacterial wilt (*Ralstonia solanacearum*), Alternaria disease, bacterial leaf spot (*Xanthomonas* sp.); 2) presence/absence of pest attacks: *Tuta absoluta* (leaf miner), *Helicoverpa armigera, Aleyrodoidea*, Mites, mealybug (*Pseudococcus viburni*), *Nesidiocoris tenuis, Liriomyza* sp.*,* Aphids, grasshopper, *Thrips* sp.*,* caterpillars, *Spodoptera* sp.
• **Yield:** The harvest is spread over five weeks, as not all fruits ripened at the same time, with one harvest a week. At each harvest, tomatoes are sorted into five categories: **category 1** - intact fruit; **category 2** - misshapen; **category 3** - eaten; **category 4** - rotten; **category 5** - holey. The tomatoes are then counted and weighed by category. Healthy tomatoes (category 1) are also sorted by size using a gauge with **diameter categories**: 1) diameter **inferior or equal to 47** mm; 2) diameter **between 47 and 57** mm; 2) diameter **between 57 and 67** mm; 3) diameter **between 67 and 82** mm; 4) diameter **between 82 and 102** mm. The tomato yields can be calculated based on the total biomass of the intact tomatoes category, the real number of plants per plot, and corrected by the theoretical planting density - 4 plants per m^2^ (32 tomato plants per plot / 8 m^2^ per plot) - and according to the Eq. (2).

### 4.7 Crops sampling and analysis

At the end of the cycle, the plants that had been monitored and measured are collected: two tomato fruits per plot at the end of each of the five harvests (10 fruits in total per plot), six lettuce rosettes per plot and eight carrot roots per plot (4 per central bed). The samples are placed in paper envelopes labelled with the treatment name and replicate number, then transported to the laboratory. The fresh biomass of lettuce leaves, carrot roots, and tomato fruits is measured on the samples. The latter are washed twice with tap water, then twice with distilled water, before being dried in an oven at 65 °C for one week. Afterwards, the dry biomass is measured using a precision electronic scale (0.1 *g*). The resulting dry biomass is ground separately using a Thomas-type knife mill (inox), to prepare for storage and analyses.

### 4.8 Samples library

All soil, crop and OWP samples collected since 2016 are stored in a Sample Bank at room temperature at the ISRA-IRD IESOL laboratory in Bel-Air, Dakar. Soil samples are stored in 180 ml polypropylene pots (approximately 250 g per sample), except for the T0 samples, stored in 1 l glass jars. The vegetable samples are stored in 50 ml polypropylene pots (approximately 20–30 g per sample). The OWP samples are stored in 500 ml plastic pots (approximately 150 g per sample), except for the digestate samples, stored in 1.5 l bottles in a freezer at −20 °C. These samples are available upon request for any measurement that might complement the available dataset.

### 4.9 Quality assurance, quality control and technical validation

**Fig. 7:**
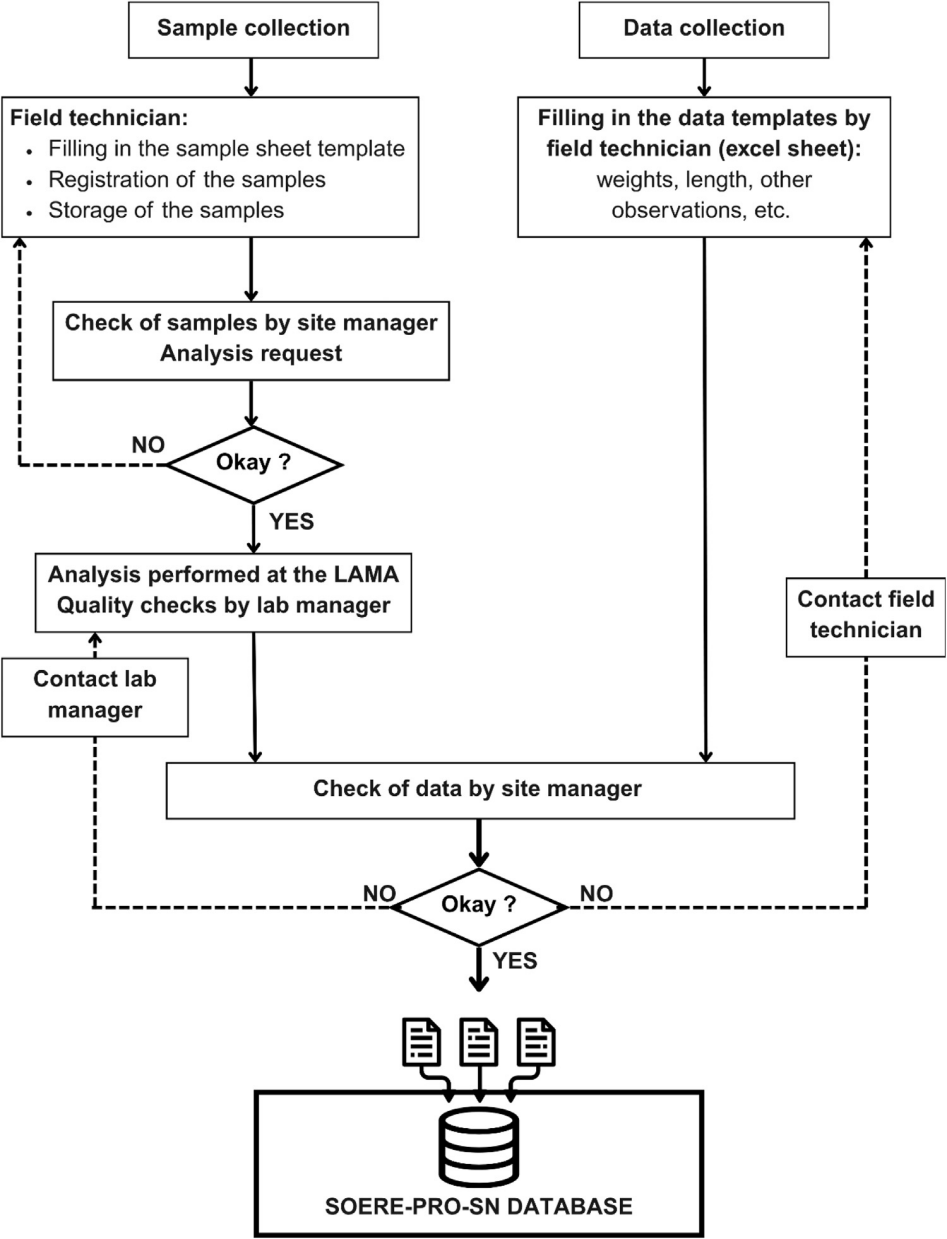
Flow chart illustrating the procedure to ensure the technical quality of the dataset.

Multiple actions are taken to ensure the quality of the dataset. Most importantly, consistent field and laboratory protocols are employed, and multiple verifications are performed at all steps. The flow chart in Fig. 7 illustrates the procedure. Data has been collected by tailored data templates which have been compiled in an iterative manner. Pre-defining experiment and treatment names, measurement variables, units and methods in the data templates reduces errors. Once collected, all samples are recorded in the laboratory inventory. The IESOL-LAMA platforms are ISO 9001 certified, ensuring full traceability of samples and proper storage conditions. In addition, a strict metrological protocol is followed to verify the accuracy of laboratory equipment. A standardized procedure is also applied to validate all analyses conducted by the LAMA laboratory in Dakar (UAR IMAGO). Before sending the results, the laboratory manager verifies the course of the analysis and confirms its reliability by examining all available data: blanks, internal and intercalibration controls and calibration curves. In case of the presence of anomalies, the manager takes required measures, including repeating the extraction, dosage or digestion process.

• **Blanks**: One blank is inserted per series (one series is maximum 31 samples). The blank must be close to zero and comparable to that of previous series. An increase may suggest contamination, while a negative value may indicate a malfunction in the analysis.
• **Internal control:** This is a sample owned by the laboratory that has been analysed many times over several years and is representative of the type of sample frequently analysed by the lab. This control is used for the pH and granulometric measurements. It allows detection of any potential drift in measurement. The results compared to previous analyses using a Z-Score (Eq. (3)), that should be within *a* ± 2 range.
  (3) $$\text{Zscore}=\frac{\left(\text{Value}\;-\;\text{control}\;\text{mean}\right)\;}{\text{control}\;\text{SD}}$$
• **Intercalibration controls**: These are samples coming from inter-laboratory exchanges (WEPAL, Wageningen Evaluating Program for Analytical Laboratories), analysed by multiple laboratories worldwide. Analysis for WEPAL is performed twice a year and all samples received for this program are kept by the LAMA. Their results help position the LAMA lab results relative to the average of those found by other laboratories. At least one intercalibration control sample is added per series). These controls are used for all analyses except for pH and granulometry. A Z-Score is calculated as described in Eq. (3) and must fall within the ±2 range.
• **Quality Control (QC):** A calibration point is prepared from stock solutions different from those used in the calibration range. Its measurement helps validate or invalidate a calibration curve.

After receiving the results, the SOERE PRO Senegal manager conducts a thorough technical validation. Each variable is plotted to detect and correct data entry errors and identify potentially erroneous measurements. Outliers are flagged and cross-checked against original field notebooks or re-analyzed when possible. Temporal consistency of the dataset is assessed by examining the trajectories of OWP, soil, and crop variables over time. Observed values and trends were consistent with known properties of sandy Arenosols in the Niayes region and with documented responses of vegetable crops to repeated organic fertilization under Sub-Sahelian conditions. Crop observations were compared with other experiments at the same site and with national variety traits. Overall, crop yields and soil nutrient concentrations fell within expected ranges, supporting the reliability and reusability of the dataset for modeling and comparative studies.

## Limitations

The dataset may be subject to random variability due to uncontrollable external factors, e.g., adverse weather (especially affecting the carrot crop), pest outbreaks or animal interferences, power cuts or water shortages, that occasionally affected harvest outcomes. When it is the case, the information is detailed in the README, for the corresponding variable.

## Ethics Statement

The authors have read and follow the ethical requirements for publication in Data in Brief and confirming that the current work does not involve human subjects, animal experiments, or any data collected from social media platforms.

## Credit Author Statement

• MLV: Project administration, Investigation, Data Curation, Validation, Writing - original draft, Writing – review and editing.
• PB: Investigation, Data Curation.
• SL: Project administration, Data Curation, Writing – review and editing.
• FD: Investigation, Data curation.
• AD: Resources.
• FF: Conceptualization, Funding acquisition, Methodology, Supervision, Project administration, Writing – review and editing.

## Data Availability

DataverseReplication Data for: A dataset of soil properties and crop observations from a long-term organic fertilization trial in Sub-Sahelian market gardening (Original data).
